# Association between mild anemia and physical fitness in a military male cohort: The CHIEF study

**DOI:** 10.1038/s41598-019-47625-3

**Published:** 2019-08-01

**Authors:** Kun-Zhe Tsai, Shiue-Wei Lai, Chia-Jung Hsieh, Chin-Sheng Lin, Yen-Po Lin, Sung-Chiao Tsai, Pei-Shou Chung, Yu-Kai Lin, Tzu-Chiao Lin, Ching-Liang Ho, Chih-Lu Han, Younghoon Kwon, Chung-Bao Hsieh, Gen-Min Lin

**Affiliations:** 1 Department of Medicine, Hualien Armed Forces General Hospital, Hualien, Taiwan; 2 Department of Medicine, Tri-Service General Hospital, National Defense Medical Center, Taipei, Taiwan; 3 Department of Public Health, Tzu Chi University, Hualien, Taiwan; 4 Department of Critical Care Medicine, Taipei Tzu Chi General Hospital, New Taipei City, Taiwan; 5 Department of Medicine, Taipei Veterans General Hospital, Taipei, Taiwan; 6 Department of Medicine, University of Virginia, Charlottesville, VA22908 USA; 7 Department of Preventive Medicine, Northwestern University, Chicago, IL 60611 USA

**Keywords:** Outcomes research, Applied physics

## Abstract

Anemia defined as reduced hemoglobin levels of red blood cells may carry less oxygen to skeletal muscle and impair physical performance. Previous studies have shown that exercise intolerance was related to moderate or severe anemia, however, the relationship to mild anemia was unknown. We investigated the cross-sectional association of mild anemia defined as a hemoglobin level of 10.0–13.9 g/dL with physical fitness in 3,666 military young males in Taiwan in 2014. Aerobic fitness was evaluated by 3000-meter run test, and anaerobic fitness was evaluated by 2-minute sit-ups and 2-minute push-ups, respectively. Multiple logistic regressions for the best 10% and the worst 10% performers were used to determine the relationship. There were 343 mild anemic males in whom 47.8% were microcytic anemia and 3,323 non-anemic males for the analysis. The multiple logistic regression shows that as compared with non-anemic males, mild anemic males were more likely to be the worst 10% performers in the 3000-meter run test (odds ratios (OR) and 95% confidence intervals: 1.47, 1.01–2.14) after adjusting for age, service specialty, body mass index, waist size, mean blood pressure, unhealthy behaviors, lipid profiles, and exercise frequency. On the contrary, mild anemic males had higher possibility to be the best 10% performers in the 2-minute push-ups test (OR: 1.48, 1.08–2.04). However, there was no association between mild anemia and 2-minute sit-ups. Our findings suggest that unspecified mild anemia might be associated with lower cardiorespiratory fitness but not with anaerobic fitness in physically active military males.

## Introduction

Anemia is characterized by a decrease in the hemoglobin levels or number of red blood cells, consequently leading to the oxygen-carrying capacity of red blood cells insufficient to meet the body’s physiologic needs. The global prevalence of anemia is high, affecting 1.93 billion people which corresponds to 27.0% of the population in 2013^1^. Iron deficiency anemia is the dominant type accounting for more than 50% of the anemic cases^1,2^. Other important causes of anemia include thalassemia trait, hemoglobinopathies, infections, chronic kidney disease, folate or vitamin B12 deficiency, gastrointestinal and gynecological conditions^3,4^. Anemia patterns may vary by age and sex^3–5^. For instance, anemia in women is much influenced by their menstruation, and in the elderly, anemia is strongly related to chronic diseases.

People with anemia could have symptoms of weakness, fatigue, poor work productivity or difficulty concentrating. The mechanisms are mainly due to a decreased maximal oxygen uptake^6^ and diminished oxygen carrying capacity, which is the major cause of reduced exercise capacity as well^7,8^. Previous reports have revealed that iron deficiency anemia may impair aerobic exercise performance by a reduction in oxygen-transport capacity of blood cells and a decrease in maximal consumption of oxygen peripherally^9,10^. In addition, iron deficiency anemia could impair mitochondrial respiratory chain activity under certain conditions, thereby limiting exercise capacity^11^.

Most of the previous studies were carried out to confirm the unfavorable effects of moderate or severe anemia, which are respectively defined as a hemoglobin of 7.0–9.9 g/dL and <7.0 g/dL on cardiorespiratory system^12,13^. However, the impact of mild anemia, defined as hemoglobin with a lower limit of 10.0 g/dL in men on physical fitness has never been examined before. Therefore, we aimed to investigate the association between mild anemia and physical performance in a large military cohort of young males who are free of chronic diseases and used to receiving regular daily rigorous training.

## Methods

### Study population

The cardiorespiratory fitness and hospitalization events in armed forces study (CHIEF) includes a historical cohort enrolling 4,080 military men and women ages between 18 and 50 years in Eastern Taiwan in 2014^14–16^. All military officers, noncommissioned officers and soldiers in Taiwan are obligated to undertake three exercise tests including 2-minute push-ups, 2-minute sit-ups, and 3000-meter run which are the same as the U.S. Army Physical Fitness Test annually^17^. All participants received the annual health surveys as well. Since moderate or severe anemia was an exclusion criterion to enlist in the Military, all of the participants had a hemoglobin level ≥10 g/dL^14–16^. Of these 4,080 participants, we excluded all 411 women in whom menstrual cycle affects the hemoglobin levels largely, and 3 men with an unexplained hemoglobin <10 g/dL, leaving a sample of 3,666 men for analysis.

The World Health Organization criteria for anemia in men and women are defined as a hemoglobin <13.0 g/dL and <12.0 g/dL, respectively^18^. Other criteria for anemia have been proposed the hemoglobin levels, ranging from 13.0 g/dL to 14.2 g/dL for men and 11.6 g/dL to 12.3 g/dL for women^19^. However, relevant criteria to clarify the value of anemia for young adults are lacking. In this study, mild anemia was defined using a relatively high upper limit of hemoglobin level of 13.9 g/dL to lower limit of a hemoglobin level of 10.0 g/dl for physically active young men and no anemia was defined as a hemoglobin level ≥14 g/dL^18,19^.

### Measurements

All military participants’ annual health examinations were carried out in the Hualien Armed Forces General Hospital of Eastern Taiwan. Each participant self-reported a questionnaire to provide details of personal medical records, including habit of cigarette smoking, alcohol consumption, betel nut chewing (current versus former or never), weekly frequency of more than 30-minutes exercise in leisure time, and medication history in the past 6 months. The annual health examination included: anthropometric measurements of height, weight, and body mass index (BMI) (weight, kg divided by square of height, m^2^; assessed in a standing position); hemodynamic data of pulse rate and blood pressures measured over right upper arm in a sitting position after a rest for 15 minutes or longer, using the FT-201 automated blood pressure monitor (Parama-Tech Co Ltd, Fukuoka, Japan). Mean blood pressure was defined as a combination of one third of systolic blood pressure level and two thirds of diastolic blood pressure (mmHg); and biochemical data of serum total cholesterol, high-density lipoprotein cholesterol, low-density lipoprotein cholesterol, fasting plasma glucose, and triglycerides concentrations analyzed enzymatically on an Olympus AU640 auto analyzer (Olympus, Kobe, Japan). Hematological parameters including white, red, and platelet blood cell counts, and levels of hemoglobin, hematocrit, and mean corpuscular volume of red blood cells were determined by the Sysmex XT- 2000-I automated hematology analyzer. All blood samples were obtained at the same blood drawing station after an overnight 8-hour fast for the participants.

### Basic physical fitness tests

The 2-minute push-up test was a measurement of anaerobic fitness superiority. The standardized 2-minute push-up test was performed on a sponge pad and recorded by a computerized monitor. The number of push-ups was scored only when the examinee’s body upward movement returned to the initial resting set level of a line of head, shoulder and buttock, as detected simultaneously by infrared sensors within 2 minutes. However, the push-ups test was aborted immediately upon either elbow or knee touching down on the sponge pad before the time ran out.

The 2-minute sit-up test was another evaluation of anaerobic fitness superiority. The 2-minute sit-up test was also performed in the similar circumstance. The examinee’s feet were fixed by the anchor on the floor with their hands close to the ears. It was scored only when the examinee’s upper body bended forward and elbows touched the electrical sensors on both thighs. If the participants violated the regulation such as the hands deviated to the ears temporarily, the test would be aborted immediately. As for the 3000-meter run, the participants ran on a flat rectangle playground at the Military Physical Training and Testing Center and did not carry any heavy object during the exercise. The test begun outdoor at 4 o’clock in the afternoon uniformly if the risk coefficient of heat stroke less than 40 (the product of outdoor temperature (°C) and relative humidity (%) × 0.1) and without heavy rainy days. All the entire test processes of each participant were recorded by video.

All eligible cases with outcome of interest were evaluated by the three exercise performances. The repetitive numbers of 2-minute push-ups mainly evaluated upper extremity muscle endurance, while the 2-minute sit-ups mainly for the endurance of abdominal muscles and hip flexors. Cardiorespiratory fitness and lower extremity muscle endurance of each participant were estimated by the time finishing a 3000-meter run. This retrospective study was approved by the Institutional Review Board of the Mennonite Christian Hospital (No. 16-05-008) in the Hualien City of Taiwan and written informed consent was obtained from all participants. We confirm that our study was performed in accordance with the 1964 Helsinki declaration and its later amendments.

### Statistical analysis

The subject characteristics were presented as mean ± standard deviation (SD) for continuous data, and numbers and percentages for categorical data. The relationship of mild anemia and performance of each exercise (i.e., numbers of 2-minute push-ups, numbers of 2-minute sit-ups and duration of 3000-m non-weight bearing running) were estimated by using analysis of covariance (ANCOVA), and the results were presented as mean ± standard error (SE). The multiple stepwise linear regression of each exercise performance with mild anemia, in reference to no anemia, was also performed. In addition, multiple logistic regression was used to determine the odds ratio (OR) of the superior (highest 10^th^ percentile) and the inferior (lowest 10^th^ percentile) performance in each exercise with mild anemia, in reference to no anemia.

A two-tailed value of p-value < 0.05 was considered significant. In model 1, age and service specialty were adjusted. In model 2, body mass index and waist size were additionally adjusted. In model 3, mean blood pressure, serum total cholesterol, triglycerides, low-density lipoprotein, alcohol intake status, betel nut chewing status, cigarette smoking status, and weekly exercise frequency were further adjusted. These potential confounders were chosen for the models based on prior published associations with physical fitness^16^ and the presence of a significant difference between the mild anemic and non-anemic groups. Statistical analyses were performed with a standard program (Statistical Package for Social Sciences, SPSS, version 25.0).

## Results

### Subject characteristics

**Figure 1:**
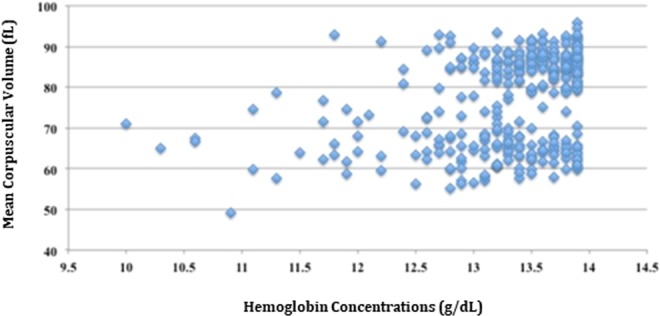
Mean Corpuscular Volume against Hemoglobin Concentrations of 343 Mild Anemic Males.

The subject characteristics of each group are shown in Table 1. The mild anemic males had greater red blood cell counts and platelet counts than the non-anemic males. Microcytic anemia (n = 164, 47.8%) accounted for nearly half of the unspecified mild anemia (Fig. 1). In addition, the mild anemic males had lower levels of BMI, waist circumference, mean blood pressure, lipid profiles, and lower prevalence of current cigarettes smoking.

**Table 1:** Subject Characteristics of the Study Cohort.

Characteristics	Mild anemia (n = 343)	No anemia (n = 3,323)	*p*-value
Age (years)	29.9 ± 0.3	29.3 ± 0.1	0.067
**Specialty**			
Army	173 [50.4]	1680 [50.6]	0.70
Navy	79 [23.0]	708 [21.3]	
Air force	91 [26.6]	935 [28.1]	
Body mass index (kg/m^2^)	24.5 ± 0.2	24.9 ± 0.1	0.019
Waist circumference (cm)	82.4 ± 0.4	83.5 ± 0.1	0.014
Mean blood pressure (mmHg)	84.7 ± 0.5	86.7 ± 0.2	<0.001
**Blood test**			
Total cholesterol (mg/dL)	165.6 ± 1.73	175.3 ± 0.65	<0.001
Serum triglyceride (mg/dL)	93.7 ± 4.1	117.3 ± 1.85	<0.001
Fasting plasma glucose (mg/dL)	92.9 ± 0.5	93.7 ± 0.2	0.30
HDL-C (mmol/L)	47.8 ± 0.5	47.9 ± 0.2	0.97
LDL-C (mmol/L)	99.5 ± 1.56	106.6 ± 0.50	<0.001
Red blood cell count (10^3^/mm^3^)	5.4 ± 0.0	5.3 ± 0.0	<0.001
Hemoglobin (g/dL)	13.3 ± 0.0	15.4 ± 0.0	<0.001
(Range: Minimum-Maximum)	(10.0–13.9)	(14.0–18.5)	
Hematocrit (%)	40.8 ± 0.1	45.7 ± 0.0	<0.001
Platelet count (10^3^/mm^3^)	263.7 ± 3.06	251.8 ± 0.95	<0.001
Mean corpuscular volume (fL)	76.9 ± 0.6	86.0 ± 0.1	<0.001
**Alcohol drinking habit**			
Never or former alcohol intake	205 [59.8]	1847 [55.6]	0.14
Current alcohol intake	138 [40.2]	1476 [44.4]	
**Betel nut chewing habit**			
Never or former chewer	311 [91.2]	2867 [87.6]	0.055
Current chewer	30 [8.8]	404 [12.4]	
**Cigarette smoking habit**			
Never or former smoker	234 [68.6]	2003 [61.2]	0.008
Current smoker	107 [31.4]	1268 [38.8]	
**Exercise frequency**			
Never or occasionally	65 [19.0]	704 [21.2]	0.37
1–2 times/week	124 [36.2]	1252 [37.7]	
≥3 times/week	154 [44.9]	1367 [41.1]	

Continuous variables are expressed as mean ± standard deviation, and categorical variables as n [%]; HDL-C, high-density lipoprotein cholesterol; LDL-C, low-density lipoprotein cholesterol.

### Group means comparisons

Table 2 shows that there was a significant difference of the 3000-meter running time between the anemic males and the non-anemic males in model 3 (861.88 sec vs. 859.60 sec, p = 0.047). However, there were similar repetitive numbers of the 2-minute push-ups and 2-minute sit-ups between the anemic males and the non-anemic males in models 1–3.

**Table 2:** Difference in Each Exercise Performance between Mild Anemic Males and Non-Anemic Males.

	2-min push-ups (numbers)			2-min sit-ups (numbers)			3000-m running (seconds)		
	n	mean ± SE	*p*-value	n	mean ± SE	*p*-value	n	mean ± SE	*p*-value
**Model 1**									
Anemia	341	50.00 ± 12.8	0.065	342	47.68 ± 8.6	0.33	311	861.87 ± 76.2	0.78
No anemia	3297	48.95 ± 11.6		3306	47.48 ± 8.1		2982	859.73 ± 72.4	
**Model 2**									
Anemia	337	50.03 ± 12.9	0.23	339	47.70 ± 8.6	0.44	308	861.88 ± 76.6	0.26
No anemia	3268	48.93 ± 11.6		3276	47.46 ± 8.1		2956	859.68 ± 71.9	
**Model 3**									
Anemia	337	50.03 ± 12.9	0.38	339	47.70 ± 8.6	0.89	308	861.88 ± 76.6	0.047
No anemia	3221	48.95 ± 11.6		3229	47.49 ± 8.1		2912	859.60 ± 72.0	

Mean (standard errors, SE) for each exercise performance estimated using analysis of covariance with adjustments for Model 1: age and service specialty adjustments; Model 2: the covariates in Model 1, body mass index and waist circumference adjustments; Model 3: the covariates in Model 2, mean blood pressure, total cholesterol, triglycerides, low-density lipoprotein cholesterol, habits of alcohol intake, betel nut chewing, cigarette smoking, and weekly exercise frequency adjustments.

### Multiple linear regressions

The results of multiple linear regressions of each exercise performance, with mild anemia relative to no anemia, in models 1–3, shown in Table 3 are in line with the findings of group means comparisons. As compared with no anemia, mild anemia was positively correlated with 3000-meter running time (β = 7.88, p = 0.047) in model 3. However, mild anemia was not correlated with numbers of 2-minute push-ups and 2-minute sit-ups in models 1–3.

**Table 3:** Liner Regression of Mild Anemia With Each Exercise Performance.

	r	β	SE	(95% CI)	*p*-value
**Model 1**					
2-min push-ups	0.14	1.25	0.67	(−0.056–2.56)	0.061
2-min sit-ups	0.26	0.44	0.45	(−0.44–1.33)	0.33
3000-m running	0.26	1.34	4.20	(−6.89–9.57)	0.75
**Model 2**					
2-min push-ups	0.28	0.75	0.65	(−0.52–2.02)	0.25
2-min sit-ups	0.29	0.31	0.45	(−0.57–1.19)	0.49
3000-m running	0.36	4.67	4.06	(−3.28–12.62)	0.25
**Model 3**					
2-min push-ups	0.32	0.57	0.64	(−0.69–1.83)	0.38
2-min sit-ups	0.34	0.063	0.44	(−0.81–0.93)	0.89
3000-m running	0.43	7.88	3.97	(0.11–15.66)	0.047

Data are presented as r and β (SE, standard errors and 95% CI, confidence intervals) using Pearson’s correlation coefficient for Model 1: age and service specialty adjustments; Model 2: the covariates in Model 1, body mass index and waist circumference adjustments; Model 3: the covariates in Model 2, mean blood pressure, total cholesterol, triglycerides, low-density lipoprotein cholesterol, habits of alcohol intake, betel nut chewing, cigarette smoking, and weekly exercise frequency adjustments.

### Multiple logistic regressions

The results of multiple logistic regressions of the best 10% and the worst 10% performances in each exercise, with mild anemia relative to no anemia, are shown in Table 4. The mild anemic males were more likely to be the best 10% performers in the 2-minute push-ups as compared to the non-anemic males in models 1–3 (OR = 1.68, 1.58, and 1.48, respectively). On the contrary, the mild anemic males had higher possibility of being the worst 10% performers in the 3000-meter run test in model 3 (OR = 1.47). However, mild anemia was not associated with the performances in 2-minute sit-ups in all models.

**Table 4:** Logistic Regression Models Comparing Mild Anemia against Non-Anemia Respectively by the Best 10% and the Worst 10% Exercise Performances.

	OR	(95% CI)	*p*-value
**Top 10% of performance level**			
**Model 1**			
2-min push-ups ≥ 60 numbers	1.68	(1.24–2.27)	0.001
2-min sit-ups ≥ 59 numbers	0.97	(0.66–1.43)	0.88
3000-m running ≤ 783 seconds	1.13	(0.85–1.49)	0.40
**Model 2**			
2-min push-ups ≥ 60 numbers	1.58	(1.16–2.16)	0.004
2-min sit-ups ≥ 59 numbers	0.94	(0.64–1.38)	0.75
3000-m running ≤ 783 seconds	1.13	(0.85–1.49)	0.39
**Model 3**			
2-min push-ups ≥ 60 numbers	1.48	(1.08–2.04)	0.015
2-min sit-ups ≥ 59 numbers	0.86	(0.58–1.28)	0.46
3000-m running ≤ 783 seconds	1.14	(0.86–1.51)	0.36
**Bottom 10% of performance level**			
**Model 1**			
2-min push-ups ≤ 37 numbers	0.95	(0.66–1.37)	0.77
2-min sit-ups ≤ 40 numbers	0.72	(0.48–1.08)	0.11
3000-m running ≥ 934 seconds	1.24	(0.86–1.78)	0.25
**Model 2**			
2-min push-ups ≤ 37 numbers	1.06	(0.73–1.54)	0.78
2-min sit-ups ≤ 40 numbers	0.76	(0.50–1.14)	0.18
3000-m running ≥934 seconds	1.38	(0.95–1.99)	0.089
**Model 3**			
2-min push-ups ≤ 37 numbers	1.08	(0.74–1.58)	0.69
2-min sit-ups ≤ 40 numbers	0.82	(0.54–1.24)	0.33
3000-m running ≥ 934 seconds	1.47	(1.01–2.14)	0.043

Data are presented as odds ratios (OR) and 95% CI (confidence intervals) using multiple logistic regression analysis for Model 1: age and service specialty adjustments; Model 2: the covariates in Model 1, body mass index and waist circumference adjustments; Model 3: the covariates in Model 2, mean blood pressure, total cholesterol, triglycerides, low-density lipoprotein cholesterol, habits of alcohol intake, betel nut chewing, cigarette smoking, and weekly exercise frequency adjustments.

## Discussion

On the basis of our findings, mild anemia might reduce the performance of short-to-medium distance running, a kind of aerobic exercise, but not affect the capacity of anaerobic exercises including short-time sit-ups and push-ups of the military males. In addition, mild anemic males had about 1.50-fold higher odds to be the best 10% performer in the 2-minute push-ups, but in contrast, to be the worst 10% performer in the 3000-meter run test. Our findings extend the concept that not only moderate or severe anemia but also mild anemia may impair the aerobic exercise capacity^12,13^.

Physiologically, the main function of red blood cells in exercise is to transport oxygen from the lungs to the peripheral tissues and deliver metabolically produced carbon dioxide to the lungs for expiration^20^. When the level of hemoglobin drops remarkably, the oxygenation of extremity skeletal muscles are reduced, possibly impairing the exercise performance in advance^12,13^. It has been reported that severe iron deficiency anemia is responsible for a decline in work capacity, particularly in aerobic endurance exercise^21,22^. Similarly as for anaerobic exercise, oxygen transport is a potent determinant of anaerobic threshold for iron deficiency anemia^23^. Therefore, anemia has been considered as a negative factor to physical fitness.

Our study shows an association between mild anemia and lower aerobic fitness. This finding confirms the concept that aerobic fitness is positively related to the overall capacity of hemoglobin in red blood cells carrying oxygen in circulation to cardiac and skeletal muscles. In addition, previous studies also reported that several physically active individuals may have anemia due to exercise-induced inflammatory cytokines production, plasma volume expansion, iron deficiency, and intravascular hemolysis from march hemoglobinuria^24–26^. As hemoglobin is consumed by the pathological reactions to overtraining, aerobic fitness could be accordingly reduced as well^27–29^.

On the contrary, mild anemia was not associated with lower anaerobic fitness. This finding could be explained by that the energy expenditure for 2-minute anaerobic exercises is oxygen free, using intramuscular adenosine triphosphate (ATP) and creatinine phosphate as the major fuel in the initial 30 seconds, and then lactate in the last 90 seconds^30,31^. Therefore, the effect of anemia on carrying oxygen would not be so critical for anaerobic fitness. In addition, the phenotype of elite long-distance runners has been found with muscle mass loss^32^, possibly reducing the intramuscular ATP and creatinine phosphate amount. In this case, the better aerobically trained individuals who had normal hemoglobin levels might have some impaired anaerobic profiles compared with the mild anemic individuals.

Our study had several strengths. First, all the exercise tests and laboratory examinations were performed in a strict manner and the procedures were standardized. Second, large numbers of military males were enrolled in this study which could provide sufficient power to detect the difference between those with and those without mild anemia. Third, only three male participants were removed from the analysis that the selection bias would be minimized. Fourth, since the daily life of military such as diet consumption and training was unified, many unmeasured confounders had been controlled at baseline. However, there were several limitations in our study. First, the study cohort included merely male subjects, making it difficult to apply the results to the female subjects. Second, the etiology of mild anemia was not fully clarified for the military members. We noticed that about half of the mild anemic males were normocytic and another 50% had a mean corpuscular volume < 80 fL suggesting a high likelihoods of iron deficiency anemia or thalassemia traits^3,33^. As occult gastrointestinal blood loss could be detected by the stool routine of the annual health examinations, iron deficiency anemia and thalassemia traits could be confirmed as the major types of mild anemia in our subjects. Third, although a number of covariates were adjusted, we could not avoid the existence of other potential confounders that may lead to a bias completely. For instance, the associations between mild anemia and physical fitness were measured merely based on a hemoglobin level from the primary dataset and no follow-up data of hemoglobin could be compared. Fourth, since the military members self-trained at their military bases, the measurement for each exercise performance was not available at baseline. However, these exercise tests are linked to the rank promotion and military award, the performance is regarded as the best physical fitness of each participant.

In conclusion, our findings suggest that unspecified mild anemia might be associated with lower cardiorespiratory fitness but not with anaerobic fitness in physically active military males. Whether the etiology of anemia may have different impacts on the exercise performances needs further investigations.
